# Stable SET knockdown in head and neck squamous cell carcinoma promotes cell invasion and the mesenchymal-like phenotype *in vitro*, as well as necrosis, cisplatin sensitivity and lymph node metastasis in xenograft tumor models

**DOI:** 10.1186/1476-4598-13-32

**Published:** 2014-02-20

**Authors:** Lays M Sobral, Lucas O Sousa, Ricardo D Coletta, Hamilton Cabral, Lewis J Greene, Eloiza H Tajara, J Silvio Gutkind, Carlos Curti, Andréia M Leopoldino

**Affiliations:** 1 Department of Clinical Analysis, Toxicology and Food Sciences, School of Pharmaceutical Sciences of Riberião Preto, University of São Paulo, Av. Café, s/n, 14040-903 Ribeirão Preto, SP, Brazil; 2 Department of Cellular and Molecular Biology, School of Medicine, University of São Paulo, Ribeirão Preto, SP, Brazil and Center for Cell Therapy and Hemotherapy of Ribeirão Preto, INCT, School of Medicine of Ribeirão Preto, University of São Paulo, Ribeirão Preto, SP, Brazil; 3 Department of Oral Diagnosis, Piracicaba Dental School, University of Campinas, Piracicaba, SP, Brazil; 4 Department of Pharmaceutical Sciences, School of Pharmaceutical Sciences of Ribeirão Preto, University of São Paulo, Ribeirão Preto, SP, Brazil; 5 Department of Molecular Biology, School of Medicine of São José do Rio Preto, Ribeirão Preto, SP, Brazil; 6 Oral and Pharyngeal Cancer Branch, National Institute of Dental and Craniofacial Research, National Institutes of Health, Bethesda, MD, USA; 7 Department of Physics and Chemistry, School of Pharmaceutical Sciences of Ribeirão Preto, University of São Paulo, Ribeirão Preto, SP, Brazil

**Keywords:** SET, Head and neck cancer, MMP, ERK, EMT, p53, Invasion, Cisplatin, Metastasis

## Abstract

**Background:**

SET/I2PP2A is a multifunctional protein that is up-regulated in head and neck squamous cell carcinoma (HNSCC). The action of SET in HNSCC tumorigenicity is unknown.

**Methods:**

Stable SET knockdown by shRNA (shSET) was established in three HNSCC cell lines: HN12, HN13, and Cal27. Protein expression and phosphorylated protein levels were determined by Western blotting and immunofluorescence, cell migration and invasion were measured by functional analysis, and PP2A activity was determined using a serine/threonine phosphatase assay. A real-time PCR array was used to quantify 84 genes associated with cell motility. Metalloproteinase (MMP) activity was assessed by zymographic and fluorometric assays. HN12shSET xenograft tumors (flank and tongue models) were established in Balb/c nude mice; the xenograft characteristics and cisplatin sensitivity were demonstrated by macroscopic, immunohistochemical, and histological analyses, as well as lymph node metastasis by histology.

**Results:**

The HN12shSET cells displayed reduced ERK1/2 and p53 phosphorylation compared with control. ShSET reduced HN12 cell proliferation and increased the sub-G1 population of HN12 and Cal27 cells. Increased PP2A activity was also associated with shSET. The PCR array indicated up-regulation of three mRNAs in HN12 cells: vimentin, matrix metalloproteinase-9 (MMP9) and non-muscle myosin heavy chain IIB. Reduced E-cadherin and pan-cytokeratin, as well as increased vimentin, were also demonstrated as the result of SET knockdown. These changes were accompanied by an increase in MMP-9 and MMP-2 activities, migration and invasion. The HN12shSET subcutaneous xenograft tumors presented a poorly differentiated phenotype, reduced cell proliferation, and cisplatin sensitivity. An orthotopic xenograft tumor model using the HN12shSET cells displayed increased metastatic potential.

**Conclusions:**

SET accumulation has important actions in HNSCC. As an oncogene, SET promotes cell proliferation, survival, and resistance to cell death by cisplatin *in vivo*. As a metastasis suppressor, SET regulates invasion, the epithelial mesenchymal transition, and metastasis.

## Introduction

SET (I2PP2A) is a 39 kDa phosphoprotein encoded by the *SET* gene. SET was originally identified as a component of the *SET-CAN* fusion gene produced by somatic translocation in acute, undifferentiated leukemia [1]. SET is a potent and specific inhibitor of protein phosphatase 2A (PP2A) [2], a serine/threonine phosphatase involved in the regulation of cell proliferation, differentiation, and transformation. SET-mediated PP2A inhibition occurs via dephosphorylation of proteins, such as the extracellular signal-regulated kinase (ERK) [3] and protein kinase B (Akt) [4]. Recently, we demonstrated that SET accumulates in head and neck squamous cell carcinoma (HNSCC) and suggested a new role for SET as a sensor of oxidative stress, thereby promoting cell survival in association with increased phosphorylated Akt levels and an enhanced antioxidant defense [5].

The mitogen-activated protein kinases (MAPKs) transduce signals from the cell membrane to the nucleus in response to a wide range of stimuli. MAPKs include three family members: ERKs (ERK1 and ERK2), c-Jun NH2-terminal kinase (JNK), and p38MAPK. ERKs are activated by phosphorylation and translocation to the nucleus where they phosphorylate multiple substrates [6]. It has been proposed that SET is a negative regulator of cell growth in response to external stimuli through inhibition of the MEK/ERK pathway and the G1/S transition [7].

The p53 protein is a tumor suppressor that protects the genome by preventing cell transformation and inducing cell cycle arrest, DNA repair, and apoptosis. p53 phosphorylation is required for signal transduction in response to DNA damage and p21 protein activation [8,9]. Indeed, SET interacts with p21 [10] and modulates p53 and Akt mRNA levels in Alzheimer’s disease neurons [11]. Of particular interest, the p53 protein is also involved in the epithelial-mesenchymal transition (EMT) [12]. The EMT promotes a mesenchymal-like phenotype in cells, that is characterized by enhanced migratory ability, invasion, and metastasis. The transmembrane protein E-cadherin is a molecular marker expressed in epithelial cells [13], and the loss of E-cadherin expression is positively correlated with tumor stage and grade. In the EMT, epithelial cells down-regulate E-cadherin and acquire mesenchymal markers, such as vimentin and fibronectin [14].

In the present study, we determined the effects of stable SET knockdown on tumorigenicity using three HNSCC cell lines, HN12, HN13 and Cal27, *in vitro* and the HN12 cell line *in vivo* (xenograft tumor models in nude mice). Our studies focused on cell invasion, proliferation, and EMT characteristics, as well as the *in vivo* xenograft tumor models, response to cisplatin, and lymphnode metastasis.

## Results

### Stable SET knockdown in the HN12 cell line decreases pERK, p-p53 and p21 expression and increases PP2A activity with a concomitant reduction in cell proliferation

**Figure 1:**
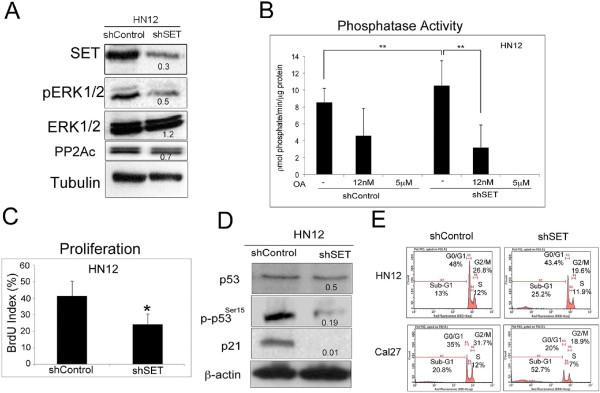
**ERK and p53 tumor-related protein levels, PP2A phosphatase activity, and proliferation and cell cycle analyses of the HNSCC shSET cells. (A)** Western blotting was performed on HN12 cells using antibodies against SET, pERK1/2, total ERK1/2 and PP2Ac; tubulin was used as a constitutively expressed protein. **(B)** PP2A phosphatase activity was determined in HN12 cells using the Ser/Thr Phosphatase Assay system in the presence or absence of the PP2A inhibitor okadaic acid (OA). **(C)** Cell proliferation was assessed in HN12 cells using the BrdU incorporation assay. **(D)** Western blotting was performed in HN12 cells using antibodies against p53, p-p53^Ser15^ and p21; β-actin was used as a constitutively expressed protein. **(E)** Cell cycle distribution was assessed by PI/flow cytometry in HN12 and Cal27 cells. The results are representative of three independent experiments. For **B** and **C**, the reported values are the mean and standard deviation of experiments performed in triplicate (*p < 0.05; **p < 0.01). For **A** and **D**, the reported values are the shSET/shControl ratio, as described in the Methods section.

HN12 cells stably expressing shRNA against SET (shSET) and control shRNA (shControl) were selected using puromycin. SET protein knockdown in HN12shSET cells (Figure 1A) has been maintained for several passages (data not shown). Using the MTS assay, HN12shSET cell viability was 88.6 ± 1.6%, and the viability of HN12 cells with siRNA for SET was 85.0 ± 2.12%.

To assess the role of SET signaling pathways in HNSCC cell survival/proliferation, we measured ERK1/2 phosphorylation (pERK 1/2, Figure 1A). Phosphorylated ERK1/2 was reduced in HN12shSET cells, suggesting that SET is involved in ERK signaling. We also used siRNA as a strategy to temporarily knock down SET protein in HN12 and Cal27 cells, and a subsequent reduction of pERK 1/2 was demonstrated (Additional file 1: Figure S1A). PP2A is an important phosphatase inhibited by SET. We observed a reduction in the PP2A catalytic (PP2Ac) subunit by Western blotting (Figure 1A); however, the serine-threonine phosphatase activity assay indicated increased PP2A activity in the HN12shSET cells compared with the HN12shControl cells (Figure 1B). Consistent with this observation, the BrdU assay indicated reduced proliferation in the HN12shSET cells compared with the HN12shControl cells (Figure 1C). Next, we determined whether the p53 protein was also modified by stable SET knockdown given that SET is reported to regulate p53 and Akt mRNA in Alzheimer’s disease [11]. Indeed, Figure 1D shows a reduction in p-p53^Ser15^ and p21 in the HN12shSET cells. In addition, p53 protein and p-p53^Ser15^ status was estimated by Western blotting in HN13 and Cal27 cell lines expressing shSET, and a reduction in phosphorylation was observed in both cell lines (Additional file 2: Figure S2). The total p53 protein level in HN13shSET cells was higher compared with control while in HN12shSET and Cal27shSET cells the level was not significantly modified. These data show that the regulation of SET is complex and suggest that each cell line may respond differently to SET knockdown. Phosphorylation of p53 at Ser-15 and Ser-20 promotes p21 protein transcription followed by cell cycle arrest at the G0/G1 phase [15]. In this regard, we observed decreases in number of G0/G1, S, and G2/M phase cells and an increase in the sub-G1 population of cells for both the HN12shSET and Cal27shSET cells compared with their respective controls (Figure 1E).

### Stable SET knockdown in HN12 cells promotes the epithelial-mesenchymal transition (EMT), cell migration, and invasion

**Figure 2:**
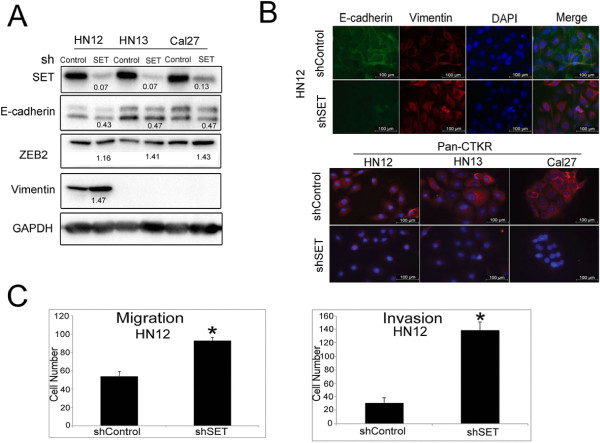
**EMT markers, cell migration and invasion analyses of the HNSCC shSET cells. (A)** Western blotting was performed for SET and the EMT markers E-cadherin, ZEB2 and vimentin in HN12, HN13 and Cal27 cells; GAPDH was used as a constitutively expressed protein. The values presented below the bands indicate the shSET**/**shControl ratio. **(B)** Immunofluorescence microscopy was used to detect E-cadherin, vimentin and pan-CTKR proteins in HN12, HN13 and Cal27 cells. **(C)** The migration assay using the chamber membrane and the invasion through matrigel were assessed in HN12shControl and HN12shSET cells. The results are either representative of three independent experiments or are reported as the means and standard deviations of experiments performed in triplicate (*p < 0.05).

The loss of p53 function is also associated with acquisition of the mesenchymal phenotype and more aggressive cancer cell migration and invasion [16]. Thus, we demonstrated that stable SET knockdown in the HN12, HN13 and Cal27 cell lines resulted in down-regulation of the epithelial marker E-cadherin and up-regulation of the EMT mediator ZEB2 (Figure 2A). The increase in the mesenchymal marker vimentin was observed only in the metastatic HN12 cells (Figure 2A); vimentin was not observed in HN13 and Cal27 cells. These data reinforce that the three cell lines studied probably represent different types of tumors. Immunofluorescence analysis confirmed the reduction of E-cadherin and the increase of vimentin in the HN12shSET cells (Figure 2B). SET knockdown using siRNA (a temporary and acute RNAi strategy) in the HN12 and Cal27 cells reduced E-cadherin level (Additional file 1: Figure S1A). In contrast to our observations using stable shSET knockdown, vimentin protein level did not increase in HN12siSET cells, suggesting that the effects in vimentin expression are chronic (Additional file 1: Figure S1A). In addition, we observed the loss of the epithelial marker pan-CTKR in the HN12, HN13, and Cal27 shSET cells (Figure 2B), illustrating the role of SET in EMT in HNSCC. Migration and invasion were studied only in the metastatic HN12 cell line and a more aggressive potential was identified in the HN12shSET cells (Figure 2C). The HN12 cells with siRNA-mediated SET knockdown displayed reduced pan-CTKR (Additional file 1: Figure S1B) and increased invasion compared with the siRNA control cells (Additional file 1: Figure S1C). This observation reinforces the view that the action of SET in the regulation of proteins and processes is related to EMT, regardless of whether SET knockdown is stable or acute/temporary. Moreover, our data for the HN12 cell line suggest that increased SET level reduces the aggressive behavior of HNSCC cells, despite the fact that increased SET typically enhances cell proliferation and survival.

**Figure 3:**
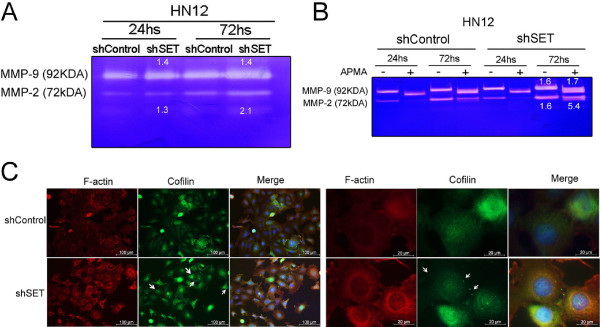
**Metalloproteinase (MMP-9 and MMP-2) activity as well as cofilin and F-actin expression in the HNSCC shSET cells. (A)** Zymogram analysis using MMP secreted from HN12shSET cells at 24 and 72 h. **(B)** Zymogram analysis was performed in the presence of 1 mM APMA for 1 h at 37°C. The values indicated below the bands represent the shSET/shControl ratio of MMP activity. **(C)** Immunofluorescence analysis of F-actin (red) and cofilin (green), demonstrating the expression and distribution pattern of F-actin and cofilin; nuclei were stained with DAPI (blue). Arrows indicate cofilin aggregates in the cytoplasm. The results are representative of three independent experiments.

We analyzed 84 genes related to cell motility by quantitative real-time PCR. Vimentin, heavy chain non-muscle myosin (MYH10), and matrix metalloproteinase 9 (MMP-9) mRNAs were up-regulated in the HN12shSET cells. In contrast, the protooncogene c-Src (SRC), Wiskott-Aldrich syndrome-like (WASL) and LIM kinase (LIMK1) mRNAs were down-regulated (Table 1). Vimentin mRNA up-regulation was accompanied by an increase in the respective protein level in HN12shSET cells (Figures 2A and 2B). The 92 kDa gelatinase B (MMP-9) and 72 kDa gelatinase A (MMP-2) were evaluated by zymogram. Increased activity was observed in the HN12shSET cells compared with control (Figure 3A), particularly when the supernatants (72 h) were activated with APMA. MMP-9 and MMP-2 were increased 1.7-fold and 5.4-fold, respectively, in HN12shSET cells (Figure 3B). The active MMP concentration was estimated by fluorometric assay, and a value of 5.89 μM was obtained for the HN12shSET cells versus 2.47 μM for the HN12shControl cells. These data support previous findings that indicate a negative correlation between ERK1/2 activation and MMP-2 activity in HNSCC tissue samples [17], suggesting that MMPs are modulated by SET in HNSCC cells.

**Table 1:** mRNA levels of 6 motility genes in the HN12shSET cells detected by real-time PCR array

**Gene**	**Fold up-and down-regulation**
	**(HN12shSET/HN12shControl)**
	Up-regulated
Vimentin	2.21
MMP-9	3.26
MYH10 (Myosin, heavy chain, non-muscle)	5.77
	Down-regulated
SRC (V-src sarcoma (Schmidt-Ruppin	−2.28
A-2) viral oncogene homolog (avian))	
WASL Wiskott-Aldrich syndrome-like	−2.28
LIMK1 (LIM domain kinase 1) -	−2.73

Cell motility is a complex and dynamic process. The cofilin protein, a regulator of actin polymerization that defines the direction of cell motility [18], is phosphorylated/inactivated by LIMK1 [19], and LIMK1 mRNA was down-regulated in the present study (Table 1). In this regard, immunofluorescence analysis using anti-F-actin and anti-cofilin in HN12shSET cells (Figure 3C) showed the cytoplasmic accumulation of F-actin and cofilin aggregates compared with control [20]. We suggest that SET knockdown reduces p21 and consequently modifies the ROCK/LIMK/cofilin pathway [21], resulting in the accumulation of F-actin and stress fibers [20]. Increased migration and invasion through the matrigel can be explained by this alteration in association with a more rapid detachment of the HN12shSET cells from the culture dish than the HN12shControl cells (data not shown) during trypsin-mediated cell detachment (4 min *vs.* 10 min, respectively), and increased MMP expression. These findings suggest that SET is involved in motility, actin dynamics (accumulation of F-actin and cofilin aggregates in cytoplasm), reduced cell adhesion, and increased MMP-9 and MMP-2 expression. Altogether, these effects confer a more aggressive phenotype to HN12shSET cells.

### Xenograft tumors from the HN12 cell line with stable SET knockdown in nude mice displayed necrosis, reduced cell proliferation, and poor differentiation

**Figure 4:**
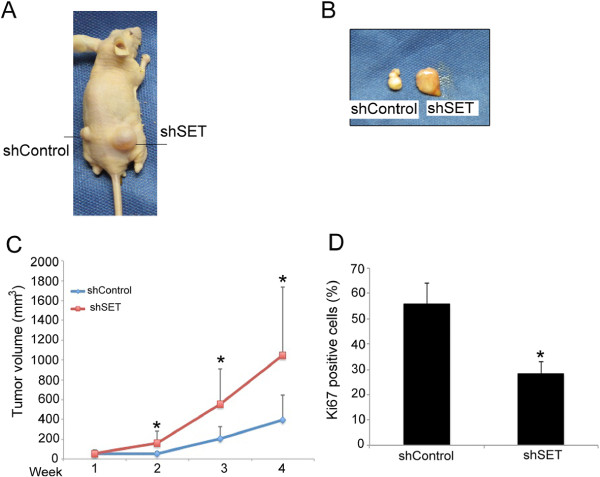
**Characteristics of the HN12shSET xenograft tumors formed in nude mice. (A)** Images of tumors formed from HN12shSET cells implanted in the flanks of Balb/c nude mice (n = 10). **(B)** The HN12shControl cells formed a solid tumor, whereas the HN12shSET tumors were similar to a large cyst and contained a yellow-white necrotic liquid that was visible when the tumors were cut. Representative images of tumors 4 weeks after cell injection are presented. **(C)** The volume of the tumors formed by HN12shSET cells. **(D)** The number of Ki67-positive tumor cells, determined as described in the Methods section. The data are reported as the means and standard deviations (*p < 0.05).

Next, we assessed the potential action of SET in tumorigenicity using HN12shSET xenograft tumors formed in Balb/c nude mice. The volume of the HN12shSET xenograft tumors was increased compared with the HN12shControl xenograft tumors (n = 10) (Figures 4A-C). In addition, the macroscopic characteristics of the tumors were different. The HN12shControl xenograft tumors (4 weeks after cell injection) contained a solid, white, homogeneous mass, whereas the HN12shSET tumor resembled a large cyst comprised of friable tissues and fluids (Figures 4B and 4C). In addition, expression of the proliferative marker Ki67 was reduced in the HN12shSET xenograft tumors compared with the control tumors (Figure 4D), in agreement with the reduced proliferative index measured *in vitro* by the BrdU assay (Figure 1C).

**Figure 5:**
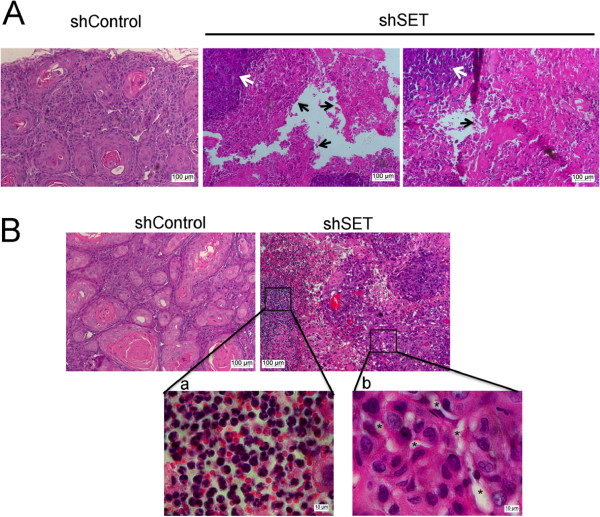
**Histological analysis of the HN12shSET xenograft tumors.** Representative images of HN12shSET xenograft tumors sections stained with H&E. **(A)** The HN12shSET cells formed a poorly differentiated tumor with reduced keratin pearl formation and liquefactive necrotic areas (black arrows), along with viable tumor cells (white arrows). **(B)** The HN12shSET tumors showed atypical mitoses, anaplastic cells and hemorrhages. The inserts show an inflammatory infiltrate **(a)** and increased blood microvessel density **(b)**. Asterisks (*) indicate microvessels.

Accordingly, histological analysis (Figure 5) demonstrated that the HN12shControl tumors were well-differentiated and produced keratin (Figure 5A). In contrast, the HN12shSET tumors were poorly differentiated and presented extensive liquefactive necrotic areas (Figure 5A). The HN12shSET tumors displayed an inflammatory infiltrate (Figure 5B-a), increased blood microvessel density (Figure 5B-b), atypical mitoses, and anaplastic cells (Figure 5B).

**Figure 6:**
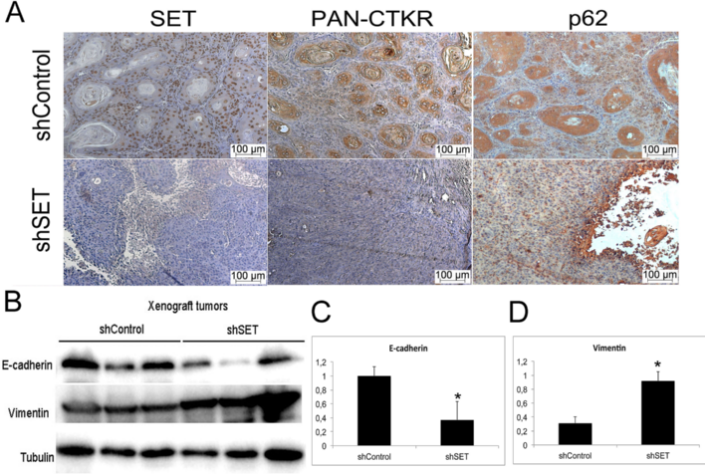
**SET, PAN-CTKR, p62, E-cadherin and vimentin expression in the HN12shSET xenograft tumors. (A)** Immunohistochemical analysis shows a reduction of SET, PAN-CTKR and p62 protein expression in the HN12shSET tumor xenografts compared with the HN12shControl tumors. **(B)** Western blotting of proteins extracted from the HN12shSET xenograft tumors. The semiquantitative analysis using densitometry confirmed reduction of the amount of E-cadherin **(C)** and increase of vimentin **(D)** protein expression compared with the shControl tumors. The bars indicate the mean and standard deviation of values obtained by densitometric analysis (*p < 0.05).

Immunohistochemistry (IHC) analysis confirmed SET protein knockdown in the HN12shSET xenograft tumor cells (Figure 6A). The HN12shSET xenograft tumor also showed a loss of pan-CTKR, indicative of poor differentiation, and reduced p62 protein (Figure 6A). In this regard, a weak p62 protein staining suggests reduced proliferation [22] and autophagy [23,24]. E-cadherin and vimentin were analyzed by Western blotting in the HN12shSET and HN12shControl xenograft tumors (Figure 6B). E-cadherin protein level was significantly reduced (Figure 6C) and vimentin protein level was increased (Figure 6D). In addition, the HN12shSET xenograft tumor showed reduced p53^Ser-15^ and ERK1/2 phosphorylation levels (Additional file 3: Figure S3).

### HN12shSET xenograft tumor models are cisplatin sensitive and display lymph node metastasis, not observed in HN12shControl xenograft tumors

**Figure 7:**
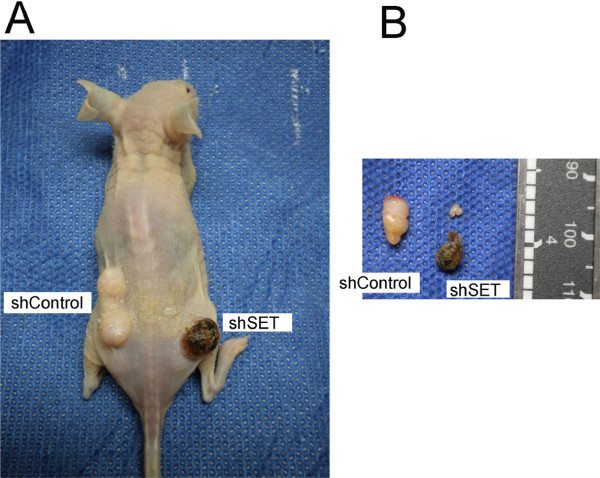
**Cisplatin sensitivity in the HN12shSET xenograft tumors.** Xenograft tumors were formed from the HN12shSET cells implanted in Balb/c nude mice as described in the Methods section. **(A)** Mice were euthanized ten days after the final cisplatin treatment (3.5 mg/kg/day, i.p., for 5 days), and **(B)** the tumors were dissected.

The potential of the SET protein as a new target in cancer therapy has been explored using a peptide [25] and sphingolipid (FTY720) [26] to disrupt the SET-PP2A interaction [27]. In the present study, we assessed SET knockdown in combination with cisplatin chemotherapy. Nude mice with HN12shSET and HN12shControl xenograft tumors (n = 5) were treated with cisplatin (Figure 7). The HN12shSET xenograft tumors were sensitive to cisplatin treatment, presenting frequent ulcerated skin lesions and tumor cell death (black tissue; Figure 7A and 7B). In contrast, the HN12shControl xenograft tumors were not affected by cisplatin treatment.

**Figure 8:**
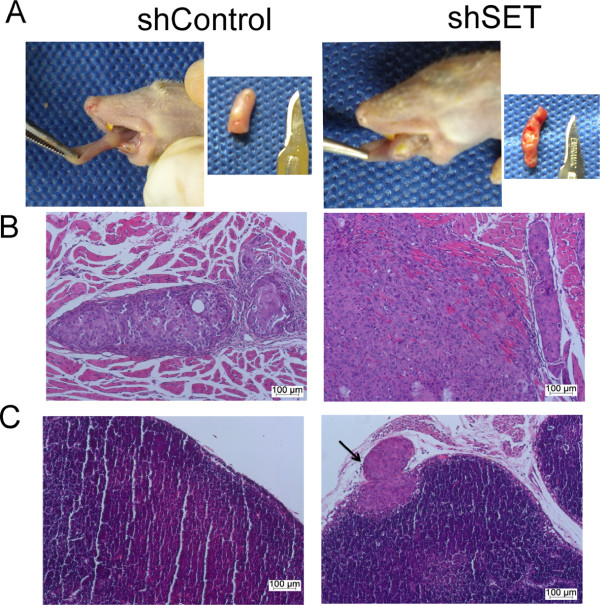
**The orthotopic HN12shSET xenograft tumor model and lymph node metastasis. (A)** Images of the tumors formed by the HN12shControl and HN12shSET cells in the tongue of Balb/c nude mice. After fifteen days, the animals were euthanized, and the tongue and lymph nodes were dissected. **(B)** Histopathological analysis of the orthotopic HN12shControl and shSET H&E-stained xenograft tumor sections. **(C)** Histopathological analysis of the cervical lymph nodes from the orthotopic HN12shSET xenograft tumor revealed the presence of metastatic cells 15 days after injection, whereas the lymph nodes in mice bearing HN12shControl xenograft tumors lacked metastatic cells.

An orthotopic tongue xenograft tumor model in nude mice (n = 3) was adopted to evaluate the metastatic potential of the HN12shSET cells compared with the HN12shControl cells. Two out of three animals were lymph node positive in the HN12shSET orthotopic tumor model, and no mice were positive in the HN12shcontrol model (Figure 8).

## Discussion

SET is associated with many cellular processes, such as cell cycle control [28], apoptosis [29], migration [30,31], DNA damage repair [32], and chromatin remodeling [33]. Recently, we demonstrated that SET extensively accumulates in HNSCC and is involved with the oxidative stress response [34]. In the present study, we addressed the effects of stable SET knockdown on tumorigenicity in HNSCC. Unexpectedly, we found that the HN12 cells became more invasive and presented a mesenchymal-like phenotype with reduced proliferation after stable SET knockdown; in addition, the HN12shSET xenograft tumors were sensitive to cisplatin chemotherapy. This is the first demonstration of the action of SET on the EMT using both *in vitro* and *in vivo* models, and these findings reinforce SET’s potential as a therapeutic target in HNSCC. Furthermore, the development of an orthotopic HN12shSET xenograft tumor model confirmed the gain of a mesenchymal-like behavior.

Previous reports have demonstrated the effects of a sphingolipid pharmacological inhibitor (FTY720) in lung cancer [26] and chronic myeloid leukemia [35] models. The antitumor action of FTY720 was associated with increased PP2A activity via specific inhibition of the nuclear SET-PP2A interaction, resulting in necroptosis [26]. The action of this pharmacological inhibitor was based on an acute condition and potent PP2A activation [27,36]. In this regard, SET could be therapeutically targeted to activate PP2A and improve tumor cell therapies. In the present study, we selected stable HNSCC cell lines with chronic SET knockdown, displaying an approximate 70% reduction in SET protein. SET knockdown was accompanied by up-regulation of PP2A activity and down-regulation of pERK and p-p53. Moreover, the mutated p53 gene has been correlated with enhanced cisplatin sensitivity via inactivation of the p53 pathway [37], consistent with our observations in the HN12shSET cells. Additionally, cisplatin cytotoxicity is associated with reactive oxygen species production [38,39], and SET was previously proposed to be a sensor of oxidative stress that promotes cell survival in HNSCC [34]. Our results suggest that SET knockdown significantly alters HN12 cell sensitivity to cisplatin-mediated death *in vivo*. Importantly, an inflammatory infiltrate and necrosis were evident in the HN12shSET xenograft tumors, suggesting that SET accumulation in HNSCC plays an important role in cell survival *in vivo*.

In numerous solid tumors, metastasis is preceded by EMT, which allows cells to repress epithelial characteristics and to acquire a mesenchymal-like phenotype that is associated with increased migration and invasion [13]. An association between p53 inactivation and the EMT, as well as a p53-mediated EMT checkpoint, has been proposed in various cancer types [12,40]. In this study, SET knockdown in HN12 cells promoted a mesenchymal-like phenotype. Moreover, SET knockdown in HN12 cells up-regulated MMP-9 and MMP-2 expression (collagenase) and altered the actin dynamic, which is important for migration and invasion [20,41]. These results suggest that SET accumulation in HNSCC promotes tumor growth while limiting cell migration and invasion. The orthotopic human xenograft tumor model showed increase of metastasis in HN12shSET cells compared with HN12shControl. Therefore, SET-mediated actions, including the classical action of increase PP2A activity that was also observed in the HN12shSET cells, may contribute to both HNSCC progression and cell differentiation *in vitro* and *in vivo*.

## Conclusions

(1) SET accumulation has important actions in HNSCC: as an oncogene, SET promotes cell proliferation, survival, and resistance to cell death by cisplatin *in vivo*; as a metastasis suppressor, SET regulates invasion, the epithelial mesenchymal transition, and metastasis. (2) This is the first time a functional response initiated exclusively by shRNA-mediated stable knockdown of SET is achieved in cancer. (3) The stable SET knockdown standardized in this study may serve as a model to evaluate the effects of SET reduction and the mechanisms related to aggressive cancer behaviors.

## Methods

The Animal Care and Use Committee of the University of São Paulo (Ribeirão Preto Campus) approved the procedures used in this study (protocol numbers 11.1.168.53.2 and 11.1.193.53.7).

### Cell culture and HNSCC cell lines with SET-knockdown

The HNSCC cell lines HN12 (tumorigenic and metastatic), HN13 [42] and Cal27 (ATCC, Manassas, VA, USA) were cultured in Dulbecco’s modified medium (DMEM, Sigma-Aldrich, Munich, Germany), supplemented with 10% fetal bovine serum (FBS, GIBCO, Carlsbad, CA, USA), antibiotics and antimycotics (Sigma-Aldrich) in a humidified atmosphere of 5% CO_2_ at 37°C. The MISSION short-hairpin RNA (shRNA) plasmid TCR1 containing DNA against human SET (shSET; TRCN0000063717; NM_003011.1-467s1c1; Sigma-Aldrich) or the shRNA control (shControl; pLKO.1puro; SHC002, shRNA non-mammalian target; Sigma-Aldrich) were transfected into HN12 cells using the Turbofect reagent (Thermo, Chelmsford, Massachusetts, USA). Stable transfectants were selected using puromycin (1 μg/mL). Lentivectors containing the shSET and shControl constructs were added to the HN13 and Cal27 cells for transduction. The transduced cells were selected using puromycin (1 μg/mL). For siRNA expression in the HNSCC cell lines, duplex RNA (siRNA) against SET was purchased from Qiagen; the protocol used for this experiment was previously reported [34]. The efficacy of SET knockdown was evaluated by Western blotting.

### Western blotting

Cell protein extracts were obtained using the CelLytic Mammalian Cell Lysis/Extraction Reagent (Sigma-Aldrich) with protease and phosphatase inhibitor cocktails (Sigma-Aldrich). Protein concentration was determined using the Bradford protein assay (Bio-Rad Laboratories, Hercules, CA, USA). Thirty micrograms of protein were separated by 10% sodium dodecyl sulfate-polyacrylamide gel electrophoresis (SDS-PAGE) and transferred to a PVDF membrane (GE HealthCare, Freiburg, Germany). The membranes were blocked in 5% non-fat dry milk in Tris-buffered saline containing 10% Tween 20. Antibodies against SET (E15; #sc-5655; Santa Cruz), ERK1/2 (M 5670; Sigma-Aldrich), phospho-p44/42 MAPK ERK1/2 (pERK1/2; #4377; Cell Signaling, Rockford, IL, USA), PP2Ac (PP2A catalytic subunit; #2038; Cell Signaling), p53 (7 F5; #2527; Cell Signaling), phospho-p53 (p-p53^Ser15^; #9286; Cell Signaling), p21WAF1/Cip1 (12D1; #2974; Cell Signaling), β-actin and tubulin were used. The reactions were developed using the chemiluminescent ECL Western blotting system (GE HealthCare). Densitometric analysis was performed using the ImageJ 6.4 software [43], and bands were normalized to constitutive proteins. The values are presented as the shSET/shControl ratio. For phosphorylation analysis, the phosphorylated/total protein ratio was calculated, and representative values are presented.

### Cell viability assay

The CellTiter 96® AQueous One Solution Cell Proliferation Assay (Promega, Madison, WI, USA) was used to determine cell viability according to the manufacturer’s instructions. The assays were performed in quintuplicate, and three independent biological experiments were considered. The cells were plated on 96-well plates 24 h before the addition of the MTS solution (tetrazolium compound and phenazine methosulfate). Next, the cells were incubated for 2 h, and the absorbance at 490 nm was recorded using a microplate reader (Bio-Rad).

### Cell proliferation assay

Cell proliferation was estimated using the bromodeoxyuridine (BrdU; Sigma-Aldrich) incorporation index as previously reported [44], with modifications. In addition, the mouse monoclonal anti-BrdU BU 33 antibody (cat. #2531; Sigma-Aldrich) was used, and IHC staining was performed using the *DAKO* LSAB + System-HRP kit (DAKO, Carpinteria, CA, USA). The BrdU-labeling index, reported as the percentage of cells labeled with BrdU, was determined by counting 10,000 cells from two independent reactions using the Zeiss Axiovert 40 inverted microscope and the AxioVision Rel. 4.8.2 software (Carl Zeiss, New York, USA).

### Cell cycle assay

The cell cycle was analyzed by flow cytometry using propidium iodide (PI; Sigma). Twenty-four hours after release from synchrony, the cells were maintained for an additional 24 h in DMEM 10% FBS. Next, the cells were collected and fixed in 70% ethanol. After washing in PBS and centrifugation, the cells were suspended in the PI (50 μg/mL) solution containing DNase-free RNAse (Invitrogen) and maintained for 30 min at 37°C. The assay was performed in a Guava*®* easyCyte*™* 8HT flow cytometry system, and the InCyte 2.2 software (Merck Millipore, Darmstadt, Germany) was used for acquisition and analysis.

### Migration and invasion assays

The *in vitro* cell migration and invasion assays were performed in 24-well plates (Corning, Inc., New York, USA) using modified Boyden chamber inserts with a polycarbonate filter membrane containing 8-μm pores. For the invasion assay, matrigel (BD Biosciences) was diluted 1:1 with serum-free medium and used to coat the filter membranes. The cells (1×10^5^) were suspended in 250 μl of serum-free DMEM and seeded onto the upper compartment of the transwell chamber; DMEM containing 10% FBS was used in the lower chamber for stimulation. After a 24 h or 72 h incubation for the migration or invasion analysis, respectively, the medium in the upper chamber was removed, and the filters were fixed in 10% formalin for 15 min. The cells on the lower surface were stained with 4′, 6-diamidino-2-phenylindole (DAPI; Sigma). Five fields were photographed at ×200 original magnification using a Zeiss Axiovert 40 inverted microscope and processed using the AxioVision Rel. 4.8.2 software. The cells were counted using the ImageJ program [43]. The migration and invasion data are reported as the number of cells per microscopic field. Five fields were analyzed.

### Quantitative real time PCR array

RNA isolation and cDNA synthesis were performed using the RNeasy and RT^2^ First Strand kits (Qiagen, Hilden, Germany), respectively. Total cDNA was used to quantify the mRNAs of 84 human genes related to cell motility in a 96-well plate array (Human Cell Motility RT^2^ Profiler™ PCR Array System, PAHS-128A-12, SABiosciences, Frederick, MD, USA) according to the manufacturer’s instructions for the Eppendorf Mastercycler EP Realplex instrument (Eppendorf, Hamburg, Germany). Up-and down-regulated genes were defined as genes with expression levels in HN12shSET cells that are >2.0 or < −2.0, respectively, [45] in relation to the HN12shControl cells. These analyses were performed in triplicate. The calculation was performed using the RT^2^ Profiler PCR Array Data Analysis, version 3.5.

### Immunofluorescence analysis

Immunofluorescence was performed with primary antibodies against E-cadherin (24E10; #3195; Cell Signaling), pan-cytokeratin (pan-CTKR; ab6401; Abcam), cofilin (#3312; Cell Signaling), F-actin (NH3; MA1-80729; Thermo-Scientific), β-actin (C4: sc-47778; Santa Cruz), and vimentin (Clone V9; V6389; Sigma). Cells were incubated with either an anti-rabbit antibody conjugated with Alexa 488 or an anti-mouse antibody conjugated with Alexa 594 (Invitrogen). The nuclei were stained with DAPI. The digital images were obtained using a Zeiss Axiovert 40 inverted microscope and processed using the AxioVision Rel. 4.8.2 software (Carl Zeiss).

### Zymographic analysis of matrix metalloproteinases

Cells (3×10^5^) were plated in a 6-well plate in triplicate. After 24 h, the medium was changed to DMEM without fetal bovine serum (FBS), and the cells were maintained for an additional 24 h and 72 h. The supernatant was collected from three wells and concentrated in an Amicon Ultra Centrifugal Filter Device 10,000 MWCO (Millipore, UFC901024). The cells were then counted. Ten microliters of concentrated supernatant was activated with 1 mM 4-aminophenylmercuric acetate (APMA; Sigma-Aldrich) for 1 h at 37°C or not treated. The samples were resolved by 12% SDS-PAGE containing 1 mg/mL gelatin. The gel was washed with 2% Triton X-100 for 40 min and incubated in a reaction buffer containing 10 mM Tris–HCl, pH 8.0, and 5 mM CaCl_2_ for 16 h at 37°C. The gel was then stained with 0.25% Coomassie blue. After removing the stain, the negative bands representing the MMP activity were visualized. Semiquantitative analysis using densitometry was performed with the ImageJ 6.4 software [43]. The results are reported as the shSET/shControl ratio.

### Fluorometric matrix metalloproteinase assay

The molar concentration of active matrix MMP in the cell culture supernatants was determined by active-site titration using the inhibitor phosphoramidon and the method of Klemencic et al. [46] with modifications. An inhibitor cocktail containing E-64 (cat. #E3132; Sigma-Aldrich), PMSF (cat. #78830; Sigma-Aldrich), and pepstatin (cat. #77170; Sigma-Aldrich) was used. The reaction mixture contained 1.9 mL of 30 mM Tris–HCl, pH 8.0, the inhibitor cocktail (10 μM final concentration), and the supernatant from the cell cultures. After incubation for 2 min at 37°C, the fluorogenic peptide substrate Abz-KLRSYKQ-EDDnp (25 μM) was added. Substrate hydrolysis was monitored using a spectrofluorometer model Lumina fluorescence spectrometer (Thermo Scientific) at λex = 320 nm and λem = 420 nm. The inhibitor phosphoramidon (Sigma-Aldrich) was added until total enzyme inhibition was achieved.

### Serine/threonine phosphatase assay

Threonine phosphatase-2A (PP2A) activity was measured using the Serine/Threonine Phosphatase Assay system (Promega; V2460) and the synthetic peptide RRA(pT)VA (Promega). For this assay, cells were lysed with Cellytic (Sigma) containing a protease inhibitor cocktail (Sigma), and the free phosphate was eliminated from the lysates using a Sephadex G-25 resin (Promega). For measurements of phosphatase activity, a standard phosphate curve was first constructed with 0, 100, 200, 500, 1,000 and 2,000 ρmol of phosphate. The samples (2.5 μg of total protein) were incubated with or without 12 nM or 5 μM okadaic acid for 15 minutes at room temperature. The reaction was performed by adding the PP2ase-2A 5× reaction buffer (250 mM imidazole, pH 7.2, 1 mM EGTA, 0.1% β-mercaptoethanol, 0.5 mg/ml bovine serum albumin) and the Thr phosphopeptide to the samples in a 96-well plate for 10 minutes at 30°C. The reaction was stopped by incubation with the molybdate dye for 15 minutes, and the absorbance was determined at 595 nm using a microplate reader (BioRad).

### Tumorigenicity and immunohistochemistry assays

To assess the xenograft tumor growth, 2×10^6^ HN12shControl and HN12shSET cells were injected s.c. into the left and right flanks, respectively, of ten 8-week-old Balb/C male nude mice. The tumor size was measured weekly using a caliper; the volume was reported as mm^3^ and calculated using the formula 0.5 × length × width^2^. At 4 weeks post-injection, the mice were euthanized. The tumors were collected, fixed in 10% formalin, and embedded in paraffin. Five-μm sections were stained with H&E for histological analysis. Three-μm sections were used for the immunohistochemical analysis of SET, Ki67, pan-CTKR, p62, pERK1/2, and p-p53^Ser15^ proteins as previously described [5]. The following primary antibodies were used: Ki67 (ab15580; Abcam, Cambridge, England), p62/SQSTM1 (D-3: sc-28359; Santa Cruz Biotechnologies, Santa Cruz, CA, USA), pan-CTKR, SET, pERK1/2 and p-p53^Ser15^. For Ki67 analysis, five microscopic fields were analyzed for the determination of percentage of positive cells.

### Cisplatin treatment of Balb/c nude mice bearing HN12 xenograft tumors

To evaluate cisplatin sensitivity *in vivo,* five Balb/C nude mice were injected with cells as described above. Cisplatin was administered intraperitoneally at 3.5 mg/kg/day (Platinil 50 mg/100 mL; Quiral Química do Brasil) 15 days after cell injection, and the treatment continued for 5 days. The post-treatment procedures are the same as described above.

### An orthotopic HN12 human xenograft tumor model for analysis of lymph node metastasis

We used an orthotopic human xenograft tumor model to evaluate metastatic potential. The stable SET knockdown HN12 (HN12shSET) and HN12shControl cells (2 × 10^5^) were injected into the tongues of Balb/c nude mice after anesthesia (n = 3) according to the ethical conduct outlined in the Care and Use of Animals for Experimentation of the University of São Paulo. The mice were assessed daily and weighed once a week. The mice were euthanized 15 days post-injection. The tongue and lymph nodes were collected, fixed in 10% formalin, and embedded in paraffin. Five-μm sections were stained with H&E for histological analysis.

### Statistical analysis

Statistical analysis was performed using Student’s *t* test, and the results are reported as the means ± standard deviations. P values <0.05 are considered to be statistically significant.

## Abbreviations

Akt: Protein kinase B; APMA: 4-aminophenylmercuric acetate; BrdU: Bromodeoxyuridine; DMEM: Dulbecco’s modified medium; EMT: Epithelial-mesenchymal transition; ERK: Extracellular signal-regulated kinase; FBS: Fetal bovine serum; H&E: Hematoxylin-eosin; HNSCCs: Head and neck squamous cell carcinomas; JNK: c-Jun NH2-terminal kinase; LIMK: 1LIM kinase; MAPKs: Mitogen-activated protein kinases; MMP: Matrix metalloproteinase; OSCCs: Oral squamous cell carcinomas; PAN-CTKR: Pan-cytokeratin; PBS: Phosphate-buffered saline; PI: Propidium iodide; PI3K: Phosphatidylinositol 3-kinase; PP2A: Protein phosphatase 2A; SDS-PAGE: Sodium dodecyl sulfate-polyacrylamide gel electrophoresis; shRNA: Short-hairpin RNA; SRC: c-Src; TBST: Tris-buffered saline with 10% Tween 20; WASL: Wiskott-Aldrich syndrome-like.

## Competing interests

The authors declare that they have no competing interests.

## Authors’ contributions

LMS, AML: conceived and designed the experiments; LMS, LOS and HC: performed the experiments; LMS, LOS, HC, CC and AML: analyzed the data; LMS, CC and AML: contributed reagents/materials; LMS and RDC: performed histology and pathological analyses; LMS, CC and AML: wrote the manuscript; RDC, LJG, EHT, JSG, CC and AML: revised the manuscript; All authors read and approved the final manuscript.

## Supplementary Material

Additional file 1: Figure S1 Temporary/acute SET knockdown using siRNA in HNSCC cells: reduced pERK and E-cadherin expression in HN12 and Cal27 cells, and reduced pan-CTKR as well as increased invasive ability in HN12 cells. The experiments were performed 48 h after SET siRNA transfection. (A) Western blotting using antibodies against SET, total ERK1/2, pERK1/2, E-cadherin, vimentin and tubulin (as a constitutively expressed protein). The densitometric results are presented as the shSET/shControl ratio. ERK phosphorylation is indicated as phospho-ERK/total ERK. (B) Immunofluorescence using an anti-pan-CTKR in HN12siSET cells; nuclei were stained with DAPI (blue). (C) HN12 cells with SET knockdown (HN12siSET) and negative siRNA control cells in the matrigel invasion assay. The results are either representative of three independent experiments or are reported as the means and standard deviations of experiments performed in triplicate (*p < 0.05). Click here for file

Additional file 2: Figure S2 Reduction of p-p53^Ser-15^ in HN13 and Cal27 cell lines with stable SET knockdown (shRNA). Western blotting was performed using antibodies against total p53, p-p53^Ser-15^ and tubulin. The densitometric values represent the shSET/shControl ratio. Tubulin was used as a constitutively expressed protein. Click here for file

Additional file 3: Figure S3 Reduction of p-p53 and pERK in the HN12shSET xenograft tumors compared with HN12shControl tumors. Three-μm sections from the HN12shSET xenograft tumors were used for immunohistochemical analysis with antibodies against p-53^Ser-15^ and pERK1/2. The images are representative of three experiments. The immunocomplexes were visualized with a chromogenic substrate (DAB; brown) and counterstained with hematoxylin. Click here for file
