# Supplementary figures and images for: Stable SET knockdown in head and neck squamous cell carcinoma promotes cell invasion and the mesenchymal-like phenotype in vitro, as well as necrosis, cisplatin sensitivity and lymph node metastasis in xenograft tumor models
Source: Mol Cancer. 2014 Feb 20;13:32. doi: 10.1186/1476-4598-13-32 (PMC3936887; doi:10.1186/1476-4598-13-32)

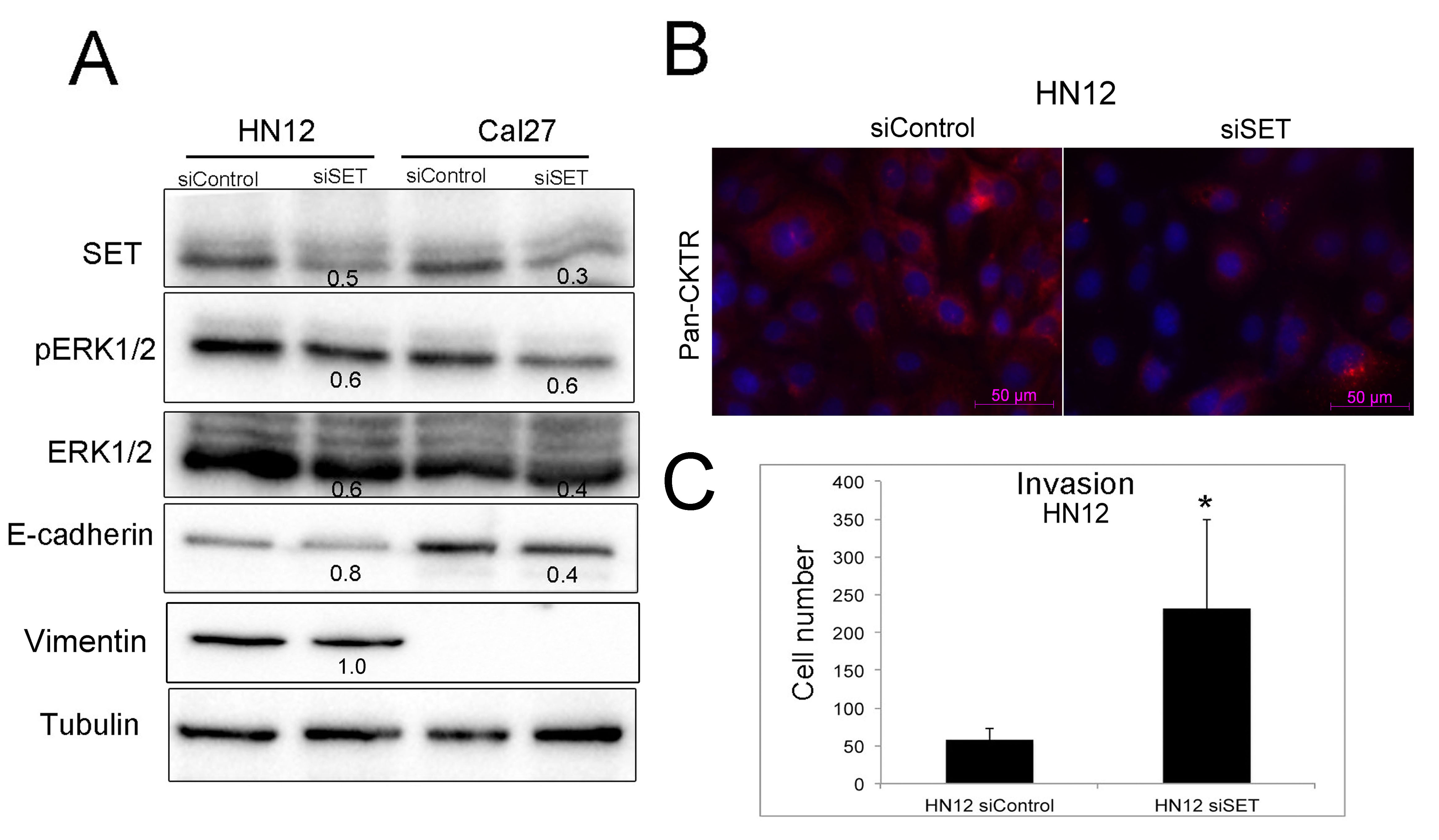

Supplement: Additional file 1: Figure S1 — Temporary/acute SET knockdown using siRNA in HNSCC cells: reduced pERK and E-cadherin expression in HN12 and Cal27 cells, and reduced pan-CTKR as well as increased invasive ability in HN12 cells. The experiments were performed 48 h after SET siRNA transfection. (A) Western blotting using antibodies against SET, total ERK1/2, pERK1/2, E-cadherin, vimentin and tubulin (as a constitutively expressed protein). The densitometric results are presented as the shSET/shControl ratio. ERK phosphorylation is indicated as phospho-ERK/total ERK. (B) Immunofluorescence using an anti-pan-CTKR in HN12siSET cells; nuclei were stained with DAPI (blue). (C) HN12 cells with SET knockdown (HN12siSET) and negative siRNA control cells in the matrigel invasion assay. The results are either representative of three independent experiments or are reported as the means and standard deviations of experiments performed in triplicate (*p < 0.05). [file 1476-4598-13-32-S1.png]

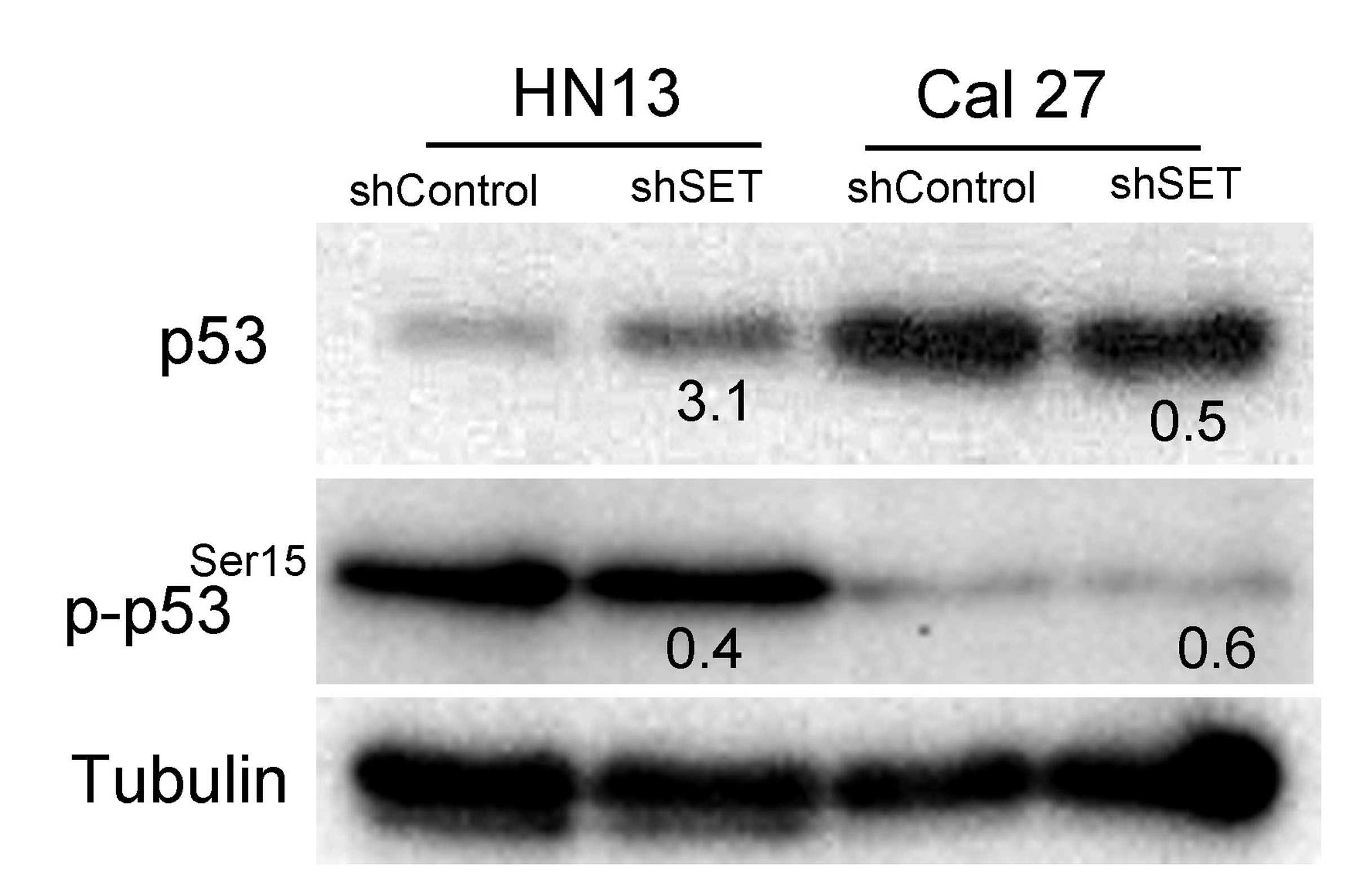

Supplement: Additional file 2: Figure S2 — Reduction of p-p53Ser-15 in HN13 and Cal27 cell lines with stable SET knockdown (shRNA). Western blotting was performed using antibodies against total p53, p-p53Ser-15 and tubulin. The densitometric values represent the shSET/shControl ratio. Tubulin was used as a constitutively expressed protein. [file 1476-4598-13-32-S2.png]

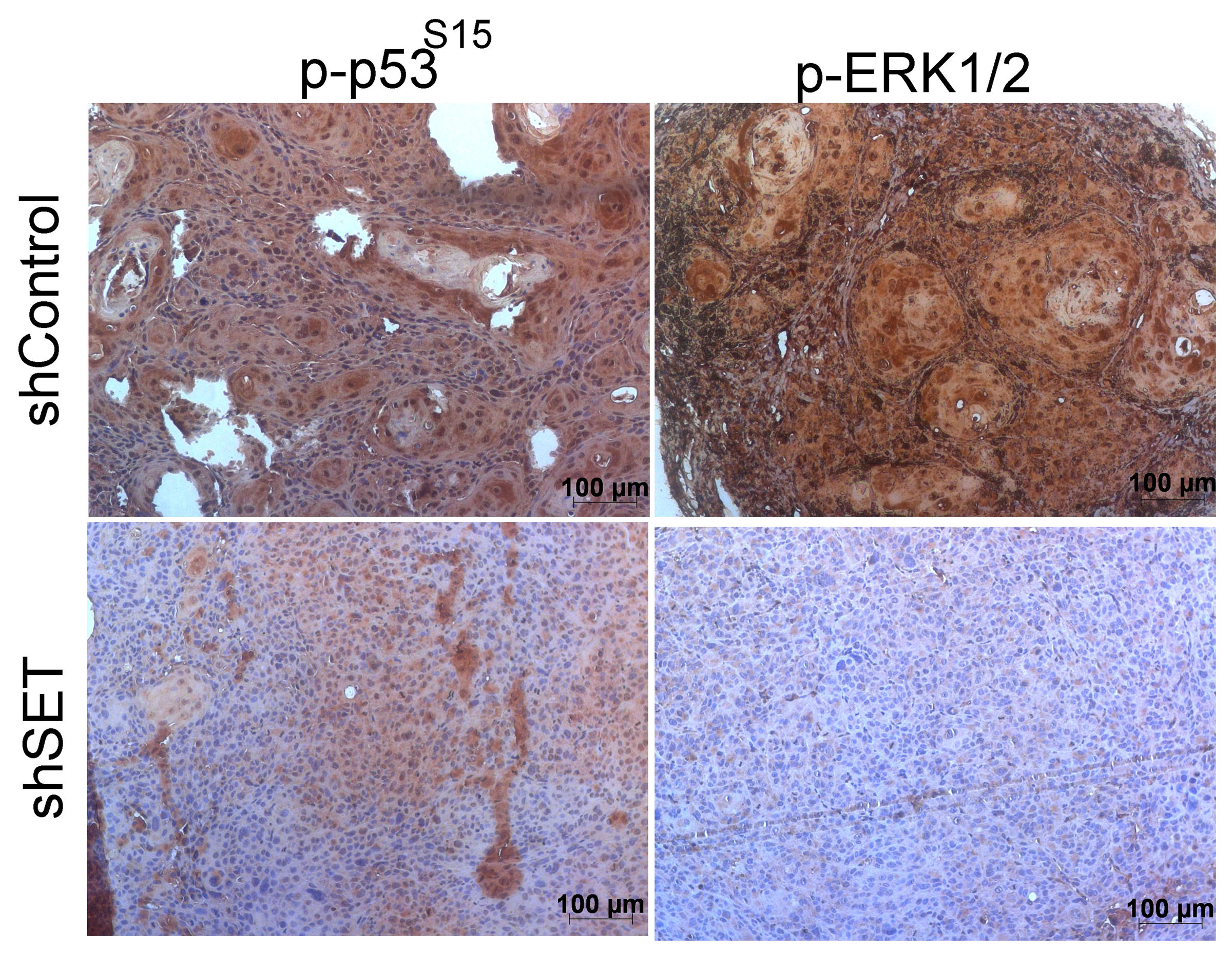

Supplement: Additional file 3: Figure S3 — Reduction of p-p53 and pERK in the HN12shSET xenograft tumors compared with HN12shControl tumors. Three-μm sections from the HN12shSET xenograft tumors were used for immunohistochemical analysis with antibodies against p-53Ser-15 and pERK1/2. The images are representative of three experiments. The immunocomplexes were visualized with a chromogenic substrate (DAB; brown) and counterstained with hematoxylin. [file 1476-4598-13-32-S3.png]
